# The moderating effect of social resources on the link between study-related stressors and depressive symptoms among medical students in North Rhine-Westphalia, Germany – a cross-sectional study

**DOI:** 10.1186/s12888-022-04170-0

**Published:** 2022-08-02

**Authors:** Nora Kappner, Jessica Lang, Anne Berthold, Petra Maria Gaum

**Affiliations:** 1 Institute for Occupational, Social and Environmental Medicine, RWTH Aachen University, Aachen, Germany; 2 Department of Health Sciences and Technology, ETH Zürich, Zürich, Switzerland

**Keywords:** Medical students, Study-related stressors, Strain, Depression, Social support, Social identity, Dual identity, Status

## Abstract

**Background:**

Previous research has demonstrated the negative effects of study-related stressors on the mental health of medical students. It has been found that social resources such as social identity, dual identity and social support help buffer negative mental health outcomes. Notably, social status has been found to weaken the connection between stress and depressive symptoms.

Based on these findings, the present study investigates how social resources (i.e., social identity, social support, dual identity and status) mitigate the impact of study-related stressors on the mental health of medical students who carry an inordinate stress burden.

**Methods:**

The data collection was based on a questionnaire (online and paper–pencil) which was distributed to medical students in North Rhine-Westphalia, Germany. The sample (224 participants) consisted of 77.2% female and 22.8% male medical students (36.2% human medicine students (HMS) and 63.8% dental medicine students (DMS)). The questionnaire included graphical scales and standardized questionnaires. We investigated demographic data, study-related stressors (i.e. academic performance, clinical practice, faculty relations) and depressive symptoms as outcomes, and social identity, social support, dual identity and status as moderators. The analyses were performed using SPSS 25 for Windows.

**Results:**

We found significant positive associations between study-related stressors and depressive symptoms. While dual identity as well as social support by fellow students emerged as buffers in these associations, the other social resources did not. As regards status, it was found to work as a buffer only in HMS, who typically enjoy a significantly higher status than dental medical students.

**Conclusion:**

It is only social resources such as support from fellow students and dual identity, but not other resource types, that can be effective buffers against depressive symptoms associated with study-related stressors. These findings can be used to promote students’ identities in relation to both fellow students and the faculty, or the university as a whole, enabling students to better cope with stress and, thus, suffer less from depressive symptoms. Furthermore, the HMS, who ascribe a relatively high status to themselves, can use their status as a buffering factor in stressful situations, in which little can be done from the outside.

**Supplementary Information:**

The online version contains supplementary material available at 10.1186/s12888-022-04170-0.

## Background

Depression, which along with anxiety disorders is one of the most widespread mental illnesses worldwide, manifests itself in symptoms such as sadness, lack of interest, hopelessness and even suicidal thoughts. According to the World Health Organization, globally, 322 million people (4.4% of the world population [1]) live with depression. In addition, depression is also a risk factor for often life-threatening diseases such as coronary heart diseases [2]. The high prevalence rate and high comorbidity potential of depression underscore the need to investigate depressive symptoms in terms of health care. Findings in Parker et al.’s study shows that 42.2% of the participants affected by depression in his study were 25 years old or younger [3]. This illness thus plays a role even at a younger age.

Studying depression among the students of a Midwestern public university, Eisenberg et al. found that a total of 13.8% undergraduates and 11.3% graduate students were positively screened for depressive symptoms [4]. This was in line with the finding by Dahlin et al. who had observed a significantly higher prevalence rate for self-rated depression in medical students (12.9%) from a medical University in Stockholm compared to a matched control group from the general population (7.8%) [5]. Being more likely to suffer from depression, medical students, compared to other students, have a higher prevalence of depression and anxiety [6]. In this study, we investigate whether study-related stressors are a reason for these higher prevalence rates. It is known that psychological stress is associated with depression in medical students [7]. After reporting on strain outcome for medical students from a Thai medical school, Saipanish found 61.4% of the 686 participants to feel stressed, with 2.4% feeling strongly stressed [8]. The participants indicated academic problems and difficulty in peer relationships, among other things, as sources of stress. The top five sources of stress in academical context were “test/exam, falling behind in reading schedule, large amount of content to be learnt, learning context – full of competition and getting poor marks”. Similar results were found in a study from Malaysia. Students cited test/examinations, large amount of context to be learnt, lack of time to review what have been learnt, getting poor marks and need to do well (self-expectation) as major sources of stress [9]. Medical students from Nepal report about the “frequency of examinations” as one of the major sources of stress [10].

Medical students can be categorized in human medicine students (HMS) and dental medicine students (DMS), with the two groups pursuing two different types of study programs. This can result in different stressors and thus in different mental health outcomes. The study programs differ among themselves and, to a lesser extent, from one university to another. Dental studies are divided into one preclinical and one clinical section with a final examination. In the case of human medicine studies, a distinction is made between the standard study program and the model study program, the main difference being that patient contact plays a role earlier in the model study program. Furthermore, the number of students in dental study programs is much smaller than in human medicine study programs, which can therefore be more anonymous. In general, it is safe to say that DMS have a greater practical component than HMS. In other European countries such as Spain and Latvia, the study programs are structured similarly. Theoretical subjects are followed by practical application in later years of study [11]. In fact, Murphy et al. report that DMS in the northeastern part of United States are more stressed in three of the five categories (academic performance, patient and clinic responsibilities, and faculty relations) than HMS [12]. Dahlin et al. report an association between academic stressors and depressive symptoms in medical students in Sweden [5] with Hamesch et al. also finding a positive correlation between study-related stressors and depression in DMS in Germany [13]. Investigating the differences between HMS and DMS and their levels of depressive symptoms, another study from Germany [14] has found DMS to have higher levels of depressive symptoms compared to HMS. Previous studies have investigated the association between stress, perceived social support and coping strategies [15]. The aim of the present study is to extend these previous findings by investigating social resources such as social support, social identity and dual identity as well as social status as possible buffering resources.

### Social identity and Social support

According to the social identity theory (SIT; [16]), social identity is the sense of belonging to a social group, which, as per the self-categorization theory (SCT; [17]), is formed by the categorization of certain characteristics. Individuals classify themselves into a corresponding group based on their own characteristics.

Another frequently studied coping strategy is seeking social support (according to the demand-control-support model; [18]). Social support from colleagues buffers symptoms of burnout as consequences of emotional and physical job demands [19]. Shao et al. have found a significant correlation between depression and social support [20], the latter being grounded in the concept of social identity. According to Haslam et al., social support is closely related to social identity; specifically, the stronger the identification with a social group (i.e. colleagues), the higher the level of perceived social support [21]. It has also been demonstrated that a high level of social identity leads to lower levels of stress. Now, it is interesting to examine whether the two factors (social identity and social support) have independent buffering effects on the association between stress and mental health outcome.

The identification with two groups at the same time is called dual identity. Simon, et al. have examined the role of dual identity in relation to political racism among migrants with questions such as ‘to which country one feels more connected; the home country or the current place of residence (namely Turkey or Germany)’ [22]. Asked whether they feel more German or Turkish, migrants have been found to identify themselves with both their home country and the country they have immigrated to.

Looking at dual identity from the aspect of stress coping potential, Welander et al. found that employees perceive more social support from their supervisors if they identify with the company [23]. However, if they identify more with their working group, they also perceived that they received more social support from their colleagues.

If the relationship between social identity and perceived social support is conflated with the idea of dual identity, it can be surmised that the identification with more than one group leads to higher levels of perceived social support.

Based on the results of Welander et al., it can be concluded that dual identity may function as a buffer against job-related stress. Employees who identify with both the organization and their immediate work group may have an easier access to resources as they are open for support: i.e. from the supervisor and from colleagues [23]. Considering dual identity as a potential buffer against job-related stress and the associated health outcomes is a new approach. Giving that the medical students who constitute our sample of interest is also nested within a university, we therefore propose they have dual identities. They belong to their study program in addition to being members of the superordinate medical faculty of their university, thus identifying with both entities. To the best of our knowledge, our study is the first to explicitly investigate dual identity in students in the context of study-related stressors and depressive symptoms. By including dual identity, we aim to test whether we can find an additional moderating benefit in the stressor-strain relationship.

### Status as buffer

In addition to investigating the social resources pertaining to social support, social identity and dual identity, we also aim to consider the role of perceived social status in the interplay between stress and depressive symptoms. Social status describes the position in a social hierarchy within groups or societies. There are two types of social status: objective and subjective. While objective social status can be measured with objective parameters (e.g., income as a proxy for socio economic status [24], subjective social status is defined as “a person’s belief about his location in a status order” [25]. Subjective social status usually depends on objective social status parameters such as household income or employment grade [26]. In a comparison between different professions in terms of occupational prestige, the highest prestige is attributed to physicians (e.g. [27]). Here, occupational prestige has been used as an indicator for the social status attributed to an occupation as part of objective status. Asked to rank various professions according to status, college students have also been found to attribute the highest status to medical professions [28] and physicians [29]. With regard to health outcomes, social status is relevant since many studies report a link between low social status and negative health issues. Subjective social status is usually a positive predictor of self-reported health in the general population even when controlling for age, gender, race, negative affect and other health risks [30]. Conversely, low subjective social status has been shown to be associated with higher rates of morbidity [26] as measured by depressive symptoms and other measures. Both men and women have been found to suffer from more severe depressive symptoms when they have low subjective social status compared to when they have high subjective social status.

In men and women aged 52 years and older, Demakakos et al. have studied the extent to which several health outcomes depend on subjective social status [24]. Their results show that a higher subjective social status is associated with a lower prevalence rate of depressive symptoms in both men and women. Subjective social status has also been found to be a negative predictor for depression in undergraduate students in their first year at university [31]. Gruenewald et al. showed subjective social status as a moderator in the relation between social threat and cortisol responses. Contrary to their expectations, no significant moderation was found [32]. In the present study, we propose to examine whether subjective social status has any buffering effect with respect to the association between study-related stress and mental health in medical students.

Considering the high prevalence of depression in medical students and the impact of social resources on health outcome, the study aims to investigate, in two steps, the social resources that influence the association between study-related stressors and mental health. First, we will analyze whether there is a direct association between the study-related stressors (described below) and mental health. Second, the moderating effect of four social resources (i.e., social support, social identity, dual identity and status) on this direct association will be investigated.

**Fig. 1:**
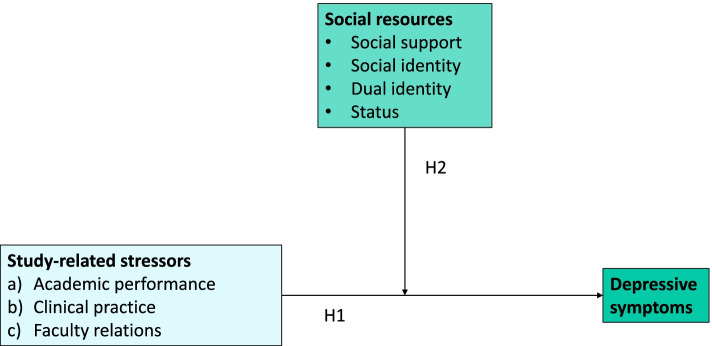
Presentation of the hypotheses. Note: H1 = hypothesis 1; H2 = hypothesis 2

In the first set of hypotheses, we assume positive direct relations between the relevant study-related stressors (academic performance (hypothesis 1a), clinical practice (hypothesis 1b) and faculty relations (hypothesis 1c)) and the health outcome pertaining to depressive symptoms (Fig. 1). In other words, the more students report study-related stressors, the more they are likely to report depressive symptoms. Moreover, we propose that social support, social identity, dual identity and status function as moderating factors that mitigate the negative consequences of study-related stressors in HMS and DMS. We postulate similar moderating effects of the 3 study-related stressors: academic performance (hypothesis 2a), clinical practice (hypothesis 2b) and faculty relations (hypotheses 2c) (Fig. 1). All hypotheses have been tested with all medical students.

**Fig. 2:**
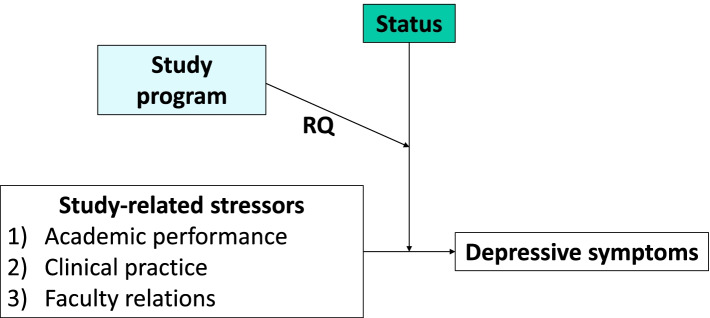
Presentation of the research question (RQ)

Considering the fact that human medicine and dentistry are different types of study programs with potentially different social status attributions, we aim to test, additionally, whether the study program influences the buffering effect of subjective social status on the association between study-related stressors (academic performance (RQ1), clinical practice (RQ2) and faculty relations (RQ3)) and depressive symptoms separately for HMS and DMS (Fig. 2). We postulate a difference in social status between the two groups with the HMS being expected to state higher status compared to the DMS. We expect the difference in status between the two study programs based on existing literature. Lehman and Witty surveyed children ages 8–18, which occupations are mostly respected in their opinion. A distinction was made between different professional groups, and physicians and dentists were also considered separately. The results show the highest respect for doctors; dentists are rated lower [33]. A study from Australia shows that dental medicine students feel marginalized compared to human medicine students. Important factors for marginalization of dental students are: limited understanding of the role of dentistry in health care, limited collaboration in workplace and different access to resources. Also based on this study, we expected a higher social status in HMS than in DMS [34]. Additionally, we predict a stronger buffering effect in the higher status group.

## Methods

### Design and procedure

In NRW (North Rhine-Westphalia) only five universities offer both study programs, human medicine and dentistry at the same faculty. This is why we sent the link for the online version of the questionnaire to student associations of these five respective universities. We asked to distribute the link among the students. At the university of the study center, both the link to the online version of the questionnaire and paper pencil version were offered to increase participation rate. The link was provided via social media. The paper pencil questionnaire was distributed in lecture halls, labs or study rooms. The number of students participating from the different universities are listed in Table 1 with “University 1, University 2,…”. For both study programs (HMS and DMS), the data were collected over a period of three semesters from May 2018 to June 2019. The questionnaire included graphical scales to generate the level of status and standardized questionnaires.

**Table 1:** Description of study population (*N* = 224)

	**N (%)**
**Gender**	
Male	51 (22.8)
Female	173 (77.2)
**Study Program**	
Human medicine	81 (36.2)
Dentistry	143 (63.8)
**University**	
1	34 (15.2)
2	9 (4)
3	2 (0.9)
4	178 (79.5)
5	1 (0.4)
**Type of questionnaire**	
Online	162 (72.3)
Paper–pencil	62 (27.7)
**Marital status**	
Living alone	158 (70.5)
Separated	4 (1.8)
Living with a partner	53 (23.7)
Married	8 (3.6)
Missing	1 (0.4)
**Depressive syndrome**	63 (28.1)

*Note*: *N* number of participants

The participants were required to provide their informed consent before answering the questionnaire. As a reward, 5 times 10€ vouchers of a big online mail order company were raffled. The study was approved by the local ethics committee (EK 139–18).

### Study population

The survey on study situation involved HMS (*N* = 78) and DMS (*N* = 146) from North Rhine-Westphalia (NRW), Germany. In total, 416 students participated (352 online and 64 on paper) in the study. The response rate of the 100 printed and distributed questionnaires was 68%, with the response rate of the online questionnaire being 36. 02%, corresponding to a total of 38.57%.

Incomplete data were not included in the analysis. A total of 178 online participants dropped out (42.8%) and 14 participants from the paper-based questionnaire had single missings. These 192 participants were excluded from the analysis, resulting in 224 participants constituting the final sample. Students from nearly all semesters were included. As those from the earlier semesters had not yet had any contact with patients, they were unable to provide any information on the clinical practice subscale. Therefore, in all analyses including this subscale, the sample size was reduced to N = 163.The final sample included 51 male (22.8%) and 173 female (77.2%) participants. Of the whole study population, only 161 made statements about their age. The mean age was 22.8 years (SD = 3.6) with a minimum of 18 years and a maximum of 35 years. The description of the final sample with all participants is in Table 1.

### Demographic data

We collected the following demographic data: University, semester, age, gender and marital status.

### Variables

#### Study-related stressors

The study-related stressors were measured with three of the five scales of the German version of the Dental Environment Stress Questionnaire (DES [12, 13]). The translated version of the questionnaire was used to measure stress in medical students by Hamesh et al. before [13]. Additionally the questionnaire was translated by different independent translators. To ensure the validity of the translated form of the questionnaire face validity, content validity and construct validity were examined [35]. The items and response categories from the original DES questionnaires were modified to clearly differentiate study-related stressors from strain outcomes. Study-related stressors were collected with 19 items divided into three subscales: academic performance (nine items e.g., “Amount of assigned coursework [fits the time allocated to them]”), clinical practice (six items e.g. “Working on patients with poor personal hygiene”) and faculty relations (four items e.g. “Inconsistency of feedback on your work among different instructors”). Students had to report, on a five-point Likert response scale, how often the respective stressors occurred (from “never” (= 1) to “always “ (= 4)). In addition, they could indicate whether the stressor was considered “not relevant “ with respect to their studies. This was especially the case in the subscale “clinical practice” because not every student had experience in clinical practice at time of participation. These students were excluded in all analyses with the variable clinical practice.

Cronbach’s alpha was 0.70 for academic performance, 0.60 for clinical practice and 0.54 for faculty relations. Mean values were computed for all three variables, with higher values indicating a respectively higher level of stress.

#### Depressive symptoms

The German version of the Patient Health Questionnaire (PHQ-D [36]) was used for measuring depressive symptoms. The scale refers to symptoms of the last two weeks and consists of nine items (e.g., “Little interest or pleasure in doing things”). Students had to indicate how often they had experienced the respective symptom on a four-point scale ranging from “not at all “ (= 0) to “almost every day “ (= 3). Cronbach’s alpha was 0.89 and a sum scale ranging from 0 to 27 was created. To detect the prevalence rate of the depressive syndrome, the items were evaluated according to the manual’s coding scheme [37] and the participants were encoded with “1” for depressive syndrome and “0” for no depressive syndrome.

#### Social resources

*Social support* was measured by means of a questionnaire of social support at work place [38]. In the response option to the items, we replaced the words superiors and colleagues with the words professors and fellow students, thus measuring social support from professors and fellow students. For example, one of the items was “how much you can rely on the following people when things get difficult during your study?”. All items had a four-point scale ranging from “not at all “ (= 1) to “absolutely “ (= 4). Cronbach’s alpha for support by professors or lectures was 0.83 and by fellow students was .91. Each mean scale was used in the analyses.

As an additional social resource, *social identity* was measured with the Group Identification Scale [39], which is a four-item scale (e.g. “I feel a connection to other human medicine students/dental medical students”) with answers ranging from “I do not agree “ (= 1) to “I absolutely agree “ (= 7). Cronbach’s alpha was 0.92. A mean scale was created.

*Dual identity* was measured with a scale adapted from Simon et al. and Simon and Ruhs [22, 40]. This scale was originally developed for people with a migration background and with more than one nationality. For the purposes of this study, we adapted the items to HMS/DMS and students of the university's medical faculty. The scale consists of four items (e.g., “I feel connected to the dental students as well as to other students of the medical faculty of my university”) with a five-point scale ranging from “not true” (= 1) to “absolutely true” (= 5). Cronbach’s alpha was 0.67 and a mean scale was formed.

Finally, *subjective social status* of the respective student group was determined by asking "How high do you estimate the status of human medicine students/dental medical students?”, respectively. Based on the approach of Adler et al., who used a ladder as a graphical scale for measuring subjective social status [41], we used a bipolar visual analog scale ranging from 0 (“low status”) to 100 (“high status”).

The description of all relevant variables is presented in Table 2. As regards status, HMS were judged to have a significantly higher status than DMS [t(195.8) = -4.8, *p* < 0.001].

**Table 2:** Description of all relevant variables (*N* = 224, *N* = 163 for clinical practice)

	**M (SD)**	**Md**	**range**	**reference**
**Study-related stressors**				1 – 4
Academic performance	2.7 (0.4)	2.6	1.2 – 3.75	
Clinical practice	2.6 (0.4)	2.7	1.5 – 3.33	
Faculty relations	2.4 (0.4)	2.3	1 – 3.75	
**Health outcome**				
Depressive symptoms	8.4 (6.0)	7.0	0—27	0—27
**Social support**				
Professors	2.0 (0.5)	2.0	1 – 3.8	1 – 4
Fellow students	3.3 (0.6)	3.4	1.4—4	1 – 4
**Social identity**	5.1 (1.4)	5.3	1 – 7	1—7
**Dual identity**	3.0 (0.8)	3.0	1 – 5	1—5
**Status**	75.8 (19.0)	81.0	1 – 100	0—100
DMS	71.1 (21.0)	72.0	1 – 100	0—100
HMS	82.2 (14.3)	85.0	2 – 100	0—100

Note: *N* number of participants, *M* mean, *SD* standard deviation, *Md* median, *DMS* dental medicine students, *HMS* human medicine students

### Statistical analyses

First, Spearman’s rank correlations were calculated between possible confounding variables and the relevant considered variables in this study (Table 3). We used Spearman’s rank correlations because not all variables had normal distribution. Gender was correlated with both the dependent and independent variables; therefore, we controlled for gender in all subsequent analyses. Although age and semester were also possible confounding variables, they could not be used as control variables because of a high amount of missing values. The first hypotheses were tested with multiple linear regression using the three study-related stressors and gender as predicting variables and the health outcome depressive symptoms as criterion variable.

**Table 3:** Spearman’s rank correlation coefficients (*N* = 224)

	gender^1^	Academic performance	Clinical practice	Faculty relations	Depressive symptoms	Social support professor	Social support fellow students	Social identity	Dual identity
Academic performance	-.22 ^**^	-							
Clinical practice	-.31 ^**^	.38 ^**^	-						
Faculty relations	-.03	.45 ^**^	.32 ^**^	-					
Depressive symptoms	-14 ^*^	.43 ^**^	.36 ^**^	.38 ^**^	-				
Social support professors	.001	-.22 ^**^	-.22 ^**^	-.38 ^**^	-.25 ^**^	-			
Social support fellow students	.01	-.37 ^**^	-.28 ^**^	-.27 ^**^	-.36 ^**^	.26 ^**^	-		
Social identity	.03	-.20 ^**^	-.16 ^*^	-.16 ^*^	-.22 ^**^	.23 ^**^	.40 ^**^	-	
Dual identity	.06	-.25 ^**^	-.20 ^*^	-.20 ^**^	-.24 ^**^	.11	.21 ^**^	.25 ^**^	-
Status	-.002	-.30 ^**^	-.19 ^*^	-.18 ^**^	-.07	.00	.17 ^*^	.13 ^*^	.24 ^**^

*Notes*: *p*-value (significance; two-tailed): +  = .05 < … < .1

^*^ *p*-value < .05

^**^ *p*-value < .01; academic performance, clinical practice and faculty relations were measured as study-related stressors; ^1^ female = 1, male = 2

To test the moderation hypotheses in the second part of this study, multiple linear regressions were again performed for each interaction hypothesis, using the SPSS macro PROCESS v 3.5 [42]. Study-related stressors were included as predictor, depressive symptoms as outcome and social resources as moderating variables.

To test our research question about subjective social status being a buffer in the group with higher perceived status, in the third part of our study, 3-term interactions were calculated with each study-related stressor, subjective social status and study program. This was done for all three study-related stressors, status and depressive symptoms depending on the study program.

All three hypotheses were further tested with a combined stress variable including all three stress scales. All results according to the combined stress variable are in a separate file [supplementary material 1].

All analyses were calculated with SPSS 25 for Windows [43]. A *p*-value of *p* < 0.05 was defined as level of significance. All hypotheses were tested for the whole study population, except the analyses including clinical practice as mentioned before.

## Results

The first hypotheses focused on the direct associations between study-related stressors (i.e., academic performance, clinical practice, faculty relations) and depressive symptoms. The multiple-linear regression shows significant positive relations between all three study-related stressors and depressive symptoms (see Table 4). The overall model was also significant [*F*(3, 159) = 17.9, *p* < 0.001, R^2^ = 0.25]. All three predictors explained 25.3% of the variance of depressive symptoms. It follows that hypothesis 1 could be confirmed for all three predictors.

**Table 4:** Results of multiple regression with study-related stressors as predictors of depressive symptoms

	*B*	S.E	β	*t*	*p*
Academic performance	**4.8**	**1.4**	**.29**	**3.5**	**.001**
Clinical practice	**2.9**	**1.3**	**.18**	**2.3**	**.02**
Faculty relations	**2.4**	**1.2**	**.16**	**2.0**	**.04**

*Notes*: Controlled for gender. *B* unstandardized regression coefficient, *S.E.* standard error, *β* standardized regression coefficient, *t* t-value, *p* p-value (significance)

In the second part of the study, the buffering effects of the four social resources on the association between study-related stressors and depressive symptoms were tested. Each study-related stressor [i.e. academic performance (H2a), clinical practice (H2b) and faculty relations (H2c)] was combined with every social resource (i.e. social support by professors and fellow students, social identity, dual identity and status). Results of each interaction term are shown in Table 5.

**Table 5:** Regression-coefficients for interactions of social resources on the association between study-related stressors and depressive symptoms

**Interactions**	**B**	**S.E**	*t*	*p*	*∆R*^*2*^
**Social support professor**					
Academic performance*social support	-1.2	1.6	-0.7	.47	.002
Clinical practice*social support	-1.7	1.9	-0.9	.38	.004
Faculty relations*social support	-1.4	0.9	-1.6	.11	.008
**Social support fellow students**					
Academic performance*social support	-1.8	1.4	-1.3	.20	.01
Clinical practice*social support	**-3.8**	**1.8**	**-2.2**	**.03**	**.02**
Faculty relations*social support	0.4	1.3	0.3	.78	.00
**Social identity**					
Academic performance*social identity	-0.3	0.6	-0.6	.56	.001
Clinical practice*social identity	-0.9	0.9	-0.9	.34	.005
Faculty relations*social identity	-0.4	0.5	-0.8	.43	.002
**Dual identity**					
Academic performance*dual identity	**-2.5**	**1.1**	**-2.2**	**.03**	**.02**
Clinical practice*dual identity	**-3.1**	**1.4**	**-2.3**	**.02**	**.03**
Faculty relations*dual identity	-0.8	1.2	-0.7	.51	.002
**Status**					
Academic performance*status	-0.05	0.1	-0.8	.40	.003
Clinical practice*status	0.04	0.1	0.5	.59	.002
Faculty relations*status	*0.1*	*0.05*	*1.8*	*.07*	*.01*

*Notes*: Controlled for gender. *N* 224, N for clinical practice = 163; *B* unstandardized regression coefficient, *S.E.* standard error, *t* t-value, *p* *p*-value (significance), ∆R^2^ = change in R^2^ (explained variance by interaction term), significant results are in bold

**Fig. 3:**
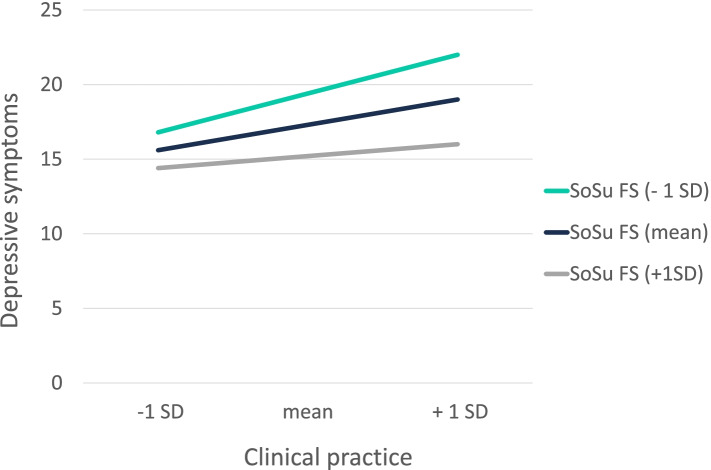
Moderating effect of social support by fellow students on the association between the study-related stressor “clinical practice” and depressive symptoms. Notes: SoSu FS = social support by fellow students; SD = standard deviation

**Fig. 4:**
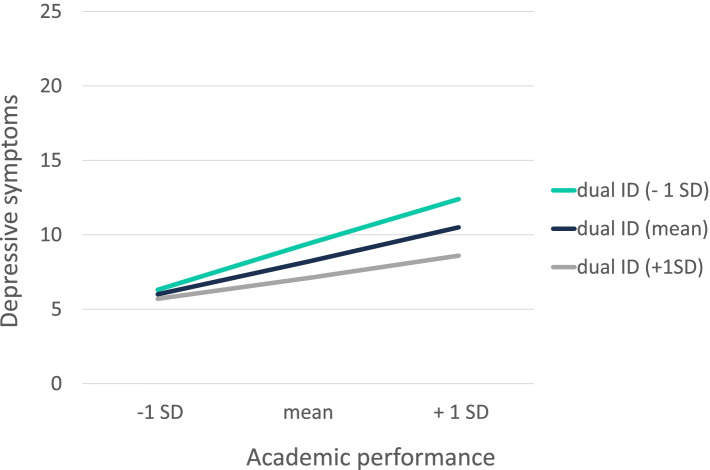
Moderating effect of dual identity on the association between the study-related stressor “academic performance” and depressive symptoms. Notes: dual ID = dual identity; SD = standard deviation

**Fig. 5:**
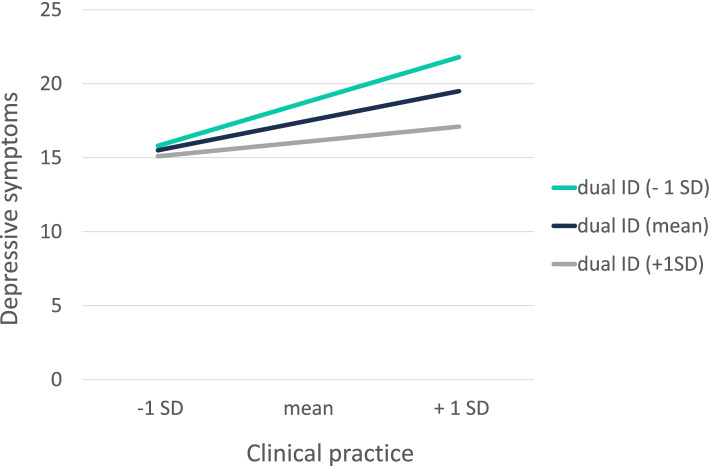
Moderating effect of dual ID on the association between the study-related stressor “clinical practice” and depressive symptoms. Notes: dual ID = dual identity; SD = standard deviation

Three significant interaction terms were found. Social support by fellow students was found to be a significant buffering factor with respect to the link between clinical practice and depressive symptoms [B = -3.8, *F(4, 158)* = *14.8*, *p* = 0.03, R^2^ = 0.02]. The visualization of the found significant interaction is illustrated in Fig. 3. The higher the report of social support from fellow students, the lower the report of depressive symptoms under higher clinical practice-related stressors. Also, dual identity was found to be a significant buffering factor with respect to the relationship between academic performance and depressive symptoms [B = -2.5, *F(4, 158)* = *16.5*, *p* = 0.03, R^2^ = 0.23] and the one between clinical practice and depressive symptoms [B = -3.1, *F(4, 158)* = *9.9*, *p* = 0.02, R^2^ = 0.20], but not for faculty relations. The observed significant interactions are illustrated in Figs. 4 and 5. The higher students reported their dual identification, the less they reported depressive symptoms under high academic performance- or clinical practice-related stressors. Thus, the buffering hypotheses can be partially confirmed. No significant interactions were found for the other social resources such as social support by professors, social identity and status.

The last part refers to our research question. As shown in the descriptive analyses (Table 1), the status of HMS was rated significantly higher than that of the DMS. In this part of the study, we sought to investigate the extent to which the study program plays a role in buffering the link between study-related stressors and depression.

**Fig. 6:**
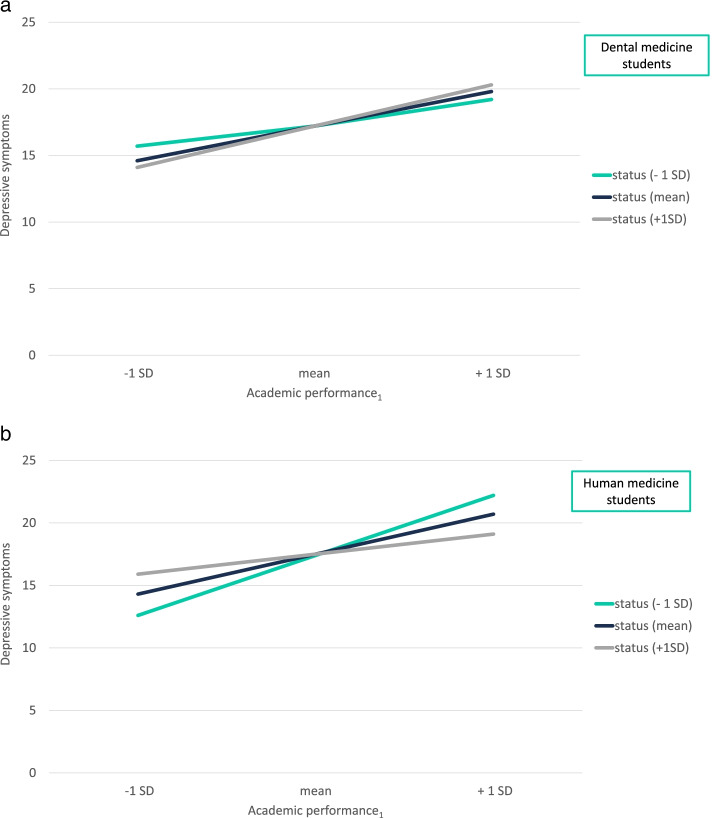
**a** Influence of ingroup status on the relationship between academic performance and depressive symptoms depending on study program. Notes: *n* = 146 dental medicine students; SD = standard deviation; _1_ = „academic performance “ was measured as study-related stressor. **b** Influence of ingroup status on the relationship between academic performance and depressive symptoms depending on study program. Notes: *n* = 78 human medicine students; SD = standard deviation; _1_ = „academic performance “ was measured as study-related stressor

Results for RQ1 show that there is a significant difference of the buffering effect of status on the relationship between academic performance and depressive symptoms for the HMS participants [B = -0.3, *F(8, 215)* = *7.1*, *p* = 0.02, R^2^ = 0.21] (Table 6). The significant interaction is illustrated in Figs. 6a and 6b. The higher the status for the HMS, the weaker is the increase in depressive symptoms when HMS report higher academic performance (Fig. 6b). The DMS do not show this buffering effect as shown in Fig. 6a. The three-way interaction was not significant for the study-related stressor “clinical practice” (RQ2) and for “faculty relations” (RQ3). Thus, the research questions could be answered only for academic performance.

**Table 6:** Results of multiple hierarchical regressions for 3-way interactions to test the research questions (RQ)

**DV: DEPRESSIVE SYMPTOMS**	**B**	**S.E**	*t*	*p*	*∆R*^*2*^
Academic performance_1_*status*study program (RQ1)	**-0.3**	**0.1**	**-2.4**	**.02**	**.02**
Clinical practice_1_*status*study program (RQ2)	*-0.3*	*0.2*	*-1.8*	*.07*	*.02*
Faculty relations_1_*status*study program (RQ3)	-0.2	0.2	-1.2	.23	.01

*Notes*: Controlled for gender. *B* unstandardized regression coefficient, *S.E.* standard error, *t* t-value, *p* p-value (significance), ∆R^2^ = change in R^2^ (explained variance by interaction term), _1_ = academic performance, clinical practice and faculty relations were measured as study-related stressor; significant results are in bold

## Discussion

We investigated the buffering effect of social resources on the relationship between study-related stressors and depressive symptoms. With respect to an additional research question, we analyzed whether subjective social status buffers study-related stressors in students of different medical study programs given that social status is differentially attributed to study programs.

Regarding the direct associations between study-related stressors and depressive symptoms (hypotheses 1), medical students (HMS and DMS) with more reported study-related stress (i.e. academic performance, clinical practice and faculty relations) also report more depressive symptoms. These results are in line with prior findings in the literature and follow the classic understanding of the stressor-strain model of stress. Similarly, Dahlin et al. report that study-related stress, measured by Higher Education Stress Inventory (HESI), leads to depressive symptoms in medical students [5]. Hamesch et al. have also demonstrated an association between study-related stressors and depression in DMS [13].

Building on the negative impact of study-related stressors on depressive symptoms, we focused on potential buffering effects of four social resources (social support, social identity, dual identity and status) on the association between study-related stressors and depressive symptoms. Interestingly, not both components of social support were found as a buffer for study-related stress. While social support from professors showed no reducing effect on depressive symptoms, social support from fellow students showed a significant effect. This observation is partially in line with prior findings. For example, Jeong and colleagues found medical students with low self-reported social support to have a tenfold higher probability for depression than their counterparts with high levels of social support [44]. Joeng et al. measured social support by family and friends and other undefined people with a standardized questionnaire. In contrast, in our study, participants were asked about organizational social support from professors and fellow students. This may account for the discrepancy in the results of these two studies given that there may be differences between receiving “professional” or study-related support from professors and fellow students and emotional support from family and friends. In contrast, Muirhead and Locker report that the greater the social support of Canadian dental students from their teachers, the lower their total stress score [45]. Hamdam-Mansour and Dawani have also found that support from friends and family leads to less stress among students in Jordan [46]. Future studies should address questions involving social support from fellow students and professors as well as from friends and family to help clarify whether the different support domains lead to different outcomes.

As for social support from professors, no buffering effect was found in this study with respect to social identity. Nevertheless, as shown in Table 3, there is a significant link between social support and social identity. Junker et al. [47] have found that a more pronounced social identity accompanies more perceived social support, which is positively related to collective self-efficacy and fewer depressive symptoms. However, in the absence of collective self-efficacy, no direct effect of social identity on depression can be detected [47]. Thus, it may be assumed that collective self-efficacy plays an important role in the association between social identity, social support and depressive symptoms. However, it is possible that collective self-efficacy was low in our study population and thus no effect was seen with respect to social support and social identity. In patients recovering from heart surgery, and in bomb disposal officers, Haslam et al. have found social identity to be positively associated with social support, thus leading to lower levels of stress [21]. According to their study [21], it can be assumed that students perceive more social support from the groups with which they identify. Self-reported subjective stress might have been another reason why no moderating effect was found for social identity. An experimental study involving students with stress triggered by means of the Trier Social Stress Test show a buffering effect of social identity only in physical stress reaction (salivary cortisol level), but not for self-reported subjective stress [48]. Future studies should also use physical indices for stress reaction to investigate the moderating effect of social identity.

Given that there were significant effects of dual identity, it is surprising that none were found with respect to social identity. In students who identify highly with both fellow students and the faculty, there is a weaker association between the stressors academic performance and clinical practice and depressive symptoms, while students with a low dual identity show stronger associations between these two study-related stressors and depressive symptoms. However, this result does not hold for the faculty relations stressor, which may be due to the fact that this stressor has a very low Cronbach's alpha. In our study, dual identity refers to the identification with students from the study program and those from the medical faculty of their university. Thus, there seems to be a difference between the people who are directly present and can provide social support and the group that forms the community of the respective study program.

As mentioned above, Welander et al. found that employees perceive more social support from the group which they identify [23]. If they identify more with their company, they perceived more social support from their supervisors, and if they identify more with their work group, they perceive more social support from their colleagues. Based on these results, it can be assumed that the identification with more than one group has the most potential as a resource for social support. Social identity and social support are highly correlated [21] and, in case of dual identity, two groups build a source for social support, likely enhancing the perception and use of social support. The results of our study indicate a weaker association of academic performance and clinical practice with depressive symptoms in students with dual identity. Medical students who identify with both their fellow students and the superordinate group, thus having two stress mitigating sources, perceive and accept support from both groups. To the best of our knowledge, this is the first study that explicitly investigates dual identity as a stress buffering moderator in the university context. Further research is necessary to strengthen the findings of this study.

The role of social status as a buffer against depressive symptoms in study-related stress was investigated in the two groups of students mentioned above. Although both groups attribute a high status to their ingroups, the results show that the HMS ascribe a higher status to themselves than DMS (Table 1). This is comparable to other findings in the literature. As already mentioned, previous studies have found students to attribute the highest status to medical professions [28] and physicians [29]. Furthermore, our results demonstrate that status is a buffering factor only in HMS, who ascribe a higher status to themselves than the DMS. However, this applies only to “academic performance” and not to all three study-related stressors we considered. Status has no influence on the relationship of the stressors clinical practice and faculty relations with depressive symptoms. Students with a higher status can use their status as a buffer, while those with a lower status lack this resource. That means, the higher the status, the higher the buffering effect of status on the correlation between academic performance and depressive symptoms. Students who do not attribute such a high status to themselves will not benefit from the resource “status”.

Now, the question that arises is, why do medical students have such a high rate of depression despite their high status. The answer lies in the fact that status has no moderating and buffering effect on the association between stress and depressive symptoms. Thus, high status by itself does not seem to reduce depressive symptoms.

Oldmeadow and Fiske report that students with a higher status ascribe themselves more competence than their counterparts with a lower status do to themselves [49]. In light of this, we interpret our results as indicating that students with a high status can use it in such a way that they can effectively buffer the stressor related to their competence. This seems to be reflected in our findings: the status can only be used as a buffer in terms of academic performance. This includes "Examination and Grades" (DES [12]). Students with a high status and therefore more self-attributed competence cope better with clinical challenges. Future studies may test status in a relationship between stress, perceived competence and depressive symptoms.

According to another study, people who feel competent in their jobs suffer from less stress and less burnout symptoms [50]. Thus, competence appears to be a helpful buffer with respect to mental health, pointing to a potentially new avenue of research in this area involving the investigation of the direct influence of competence (self-attributed or externally assessed) on mental health. Another mechanism to explain the buffering effect of status may be the association between subjective status and self-esteem. The subjective social status and self-esteem are positively correlated in students [51] and high self-esteem is a predictor of lower expression of depression [52]. The authors discuss that interventions against depression should also include the enhancement of self-esteem [52]. Status is correlated with higher self-esteem and can thus have a positive effect on depressive symptoms. According to Twenge and Campbell, there is a small but significant relationship between socioeconomic status and self-esteem [53], with people with higher socioeconomic status enjoying higher self-esteem. It is not only subjective social status that has an influence on depressive symptoms. In addition to self-esteem, appreciation may also play a role in reducing depressive symptoms. Chow and Berenbaum have found depressive symptoms to be reduced by an increase in the perceived utility of appreciation [54]. That individual aspect of appreciation lead to higher mental health has been demonstrated by Lim [55]. However, it has been shown only with respect to positive correlation, which is why it may be relevant to investigate the extent to which the lack of appreciation leads to poorer mental health. The effect of self-esteem and appreciation on depression, taking status into account, still needs to be explored.

The importance of our study is underscored by the fact that, as our sample demonstrates, 28.1% of medical students in NRW suffer from depressive symptoms, which is in line with the available literature. Eisenberg et al. show that students generally have a higher prevalence rate of depression than the world population [4]. Given that medical professions are extremely stressful [56, 57], as other studies have demonstrated, it is alarming that such a large number of students are impaired in their health even before they actually start working. In this context, a longitudinal study may shed light on any changes in the percentage of participants affected by depressive syndromes after, for example, 10 years of working life. These findings can enable universities to effectively address issues pertaining to the health of their students and ensure that they are better able to deal with study-related stressors, thus helping reduce the rate of students suffering from depressive symptoms. It is possible to expand the buffering options shown here and thereby enable others to reduce the impact of study-related stressors on their health. As shown in this study, a high subjective status can serve as a buffer in the relationship between study-related stress and depression. Accordingly, by increasing the self-perceived social status, one can also help those who do not consider their status to be high enough to reduce stress. However, if we look at the factors that determine social subjective status, namely household income or employment grade [25], we can see that little can be done about these factors from the outside. Therefore, status may not be the right starting point to buffer the connection between study-related stress and depressive symptoms. Instead, dual identity, which has shown a buffering effect for both HMS and DMS in our study, may prove useful. The university can help create an environment in which students feel comfortable enough to identify with it. At the same time, students need to be able to identify also with the community they are from. In addition to dual identity, social support from fellow students has been found have a buffering effect on the relationship between stress and depressive symptoms, whereas the same from professors has not been seen to have any mitigating effect on this association. As mentioned in the introduction, the student groups in dental school are smaller, but still too large for personal contact between professors and students. In addition, most professors themselves are fully occupied in their daily clinical routine. Maybe there is no time for closer contact. This could also be a starting point for universities to increase their students’ mental health. Increasing social support from professors could build an additional resource. As regards the strengths and limitations of our study, we only used completed data sets and excluded all those that were not complete, which allowed us to achieve relatively reliable results unaffected by possible biases caused by individual omissions. This is a strength of our study. Moreover, the data were collected only during the semester, with representative stress levels, and not during holidays.

According to Wang and Cheng, cross-sectional studies suffer from some general limitations [58], one of which is the impossibility of evaluating the temporal relationship of results; incidence is unmeasurable and making causal inferences is difficult. Only prevalence is measurable. As regards questionnaires, bias in the data may result from the fact that only a section of the representative population answers the questionnaires. The data of those who do not answer the questionnaires are therefore not taken into account and cannot be included in the results. One limitation of this study is certainly the small sample size. However, considering N sizes from studies within the same subject area (e.g. [47].), our results and sample size fit well into the existing literature. The willingness to fill out the questionnaire was very low. The assessment occurred during the semester. Perhaps this is also a sign that the students did not have much extra time to fill out the questionnaire next to their study demands. Unfortunately, the willingness to pass on the questionnaires at universities outside the study center was also very low. Also, the offering of an incentive did not seem to have elicited an excessive participation rate. At least a bias in participation for the purpose of receiving the voucher can be ruled out.

Furthermore, it is questionable whether the sample is representative of all HMS and DMS at universities in NRW, as only five universities were included, where both fields of study are offered. An additional limitation is that, out of these five universities, participants came primarily from one. In our opinion, however, this should be put into perspective to the extent that the courses at the various universities are subject to the same study regulations. Thus, there are only minor differences between the universities.

Also, the gender distribution in our study does not correspond to the average. According to German statistics, in the 2018/2019 academic year, about 35.25% of DMS and 37.95% of HMS were male [59, 60], a larger proportion in both fields of study than in our sample (22.8%). On the other hand, the gender distribution in these studies is not the same at every university and therefore does not always correspond to the average.

Another possible weakness may be the validity of the instrument to measure stress among medical students. However, our results are comparable to other studies that used the same questionnaire to elicit the stress variable [12, 13]. Adler et al. have measured socio-economic status using a graphical scale, where participants are asked to mark where they see themselves on the scale [41]. The criteria that are decisive here are money, education and occupation. On one side of the scale are those people who earn a lot of money, have a high education and a good job, and on the other side there are those who have little money, a low level of education and a bad or no job. In contrast, we have only marked the scale with “high” and “low” status. The disadvantage is that the participants may have judged the status based on different aspects. On the other hand, this is exactly what we need to discuss whether precisely these parameters would have made a difference in our sample, since both HMS and DMS are student groups from the medical field [28, 29]. Future studies involving the topic of social status should be cognizant, as Adler et al. [41], of recording status using parameters that are adequately clear.

Methodologically, the existing multicollinearity is a further limitation of this study. The fact that the predictor variables are partially correlated can make it difficult to interpret the higher order terms. To ensure the best possible interpretation, all three study-related stressors were simultaneously considered as predictors. In addition, standardized values were used for calculations of interaction terms, to prevent any computational problems from multicollinearity [61].

Another methodological aspect is the relatively low level of Cronbach's alpha values for the scales we used to measure study-related stressors and dual identity, especially for social support as mentioned above. However, it should be noted that an internal consistency of 0.60 is regarded sufficient for group-related studies [62].

According to the limitations of such a cross-sectional study, the disadvantage that the temporal development cannot be observed plays a greater role due to the emergence of the Covid-19 pandemic. Here, it would have been interesting to note the extent to which the level of depression might have changed, and whether a change in the perception of social support had occurred due to social isolation.

Since the beginning of 2020, the lives of many people have been significantly affected by the pandemic, including those of students. Some studies have looked at the mental health impact of the pandemic. In a cross-sectional study focusing on medical students and trainees, Pandey et al. show that, at the time of measurement at the beginning of the pandemic, anxiety and depression play a role in the mental health of medical students [63]. The results of the study also show a large proportion of participants to have changed social interactions with self-isolation accounting for 45.8% of them [63]. Summarizing the aspects of social isolation and loneliness in relation to the pandemic, Banerjee and Rai [64] conclude that social isolation and loneliness lead to higher rates of depression. In the context of our study, it would now be interesting to investigate the extent to which the results might have changed due to the pandemic. It is possible that some dental medicine students have not changed as much, as teaching in clinical courses may have been face-to-face and thus social isolation may not have played quite as large a role as in other professional courses.

## Conclusions

In this study, we found that there are significant positive correlations between study-related stressors such as academic performance, clinical practice and faculty relations and depressive symptoms among medical students. Furthermore, dual identity in particular acts as a significant moderator in this context, buffering depressive symptoms caused by study-related stress. Finally, students who ascribe a high status to themselves, in our study the HMS, can also use their status as a buffering factor in competence-related stress situations. However, this does not benefit the students who ascribe a lower status to themselves (DMS).

Given the high prevalence of depressive syndromes observed during the semester, it may be advisable for universities to pay closer attention to issues pertaining to the mental health of their students, as eliminating the reported stressors from the realm of medical studies may be impossible. The starting point for improving the situation may therefore lie in augmenting the social resources, which, on the part of the university, may be accomplished through professional and social support.

Future research can explore whether the difference in status between HMS and DMS results in differences in coping strategies. It would also be interesting to investigate whether students who ascribe a high status to themselves also have a higher level of self-confidence and are therefore more resistant to stress, and how the university can help increase subjectively perceived status in students. In addition to depressive symptoms, other mental health outcomes should also be evaluated.

## Supplementary Information

**Additional file 1:** **Table S1.** Spearmans`rank correlation coefficients for mean stress. **Table S2.** Regression-coefficientsfor interactions of social resources on the association between mean stressorsand depressive symptoms. 

## Data Availability

The data supporting the findings of this study are not publicly available for reasons of sensitivity, as they are human data, and may be made available by the corresponding author upon reasonable request.

## Abbreviations

B: Unstandardized Regression coefficient
β: Standardized regression coefficient
DES: Dental Environment Stress Questionnaire
DMS: Dental medicine students
H: Hypothesis
HMS: Human medicine students
M: Mean
N/n: Number of participants
NRW: North Rhine-Westphalia
p: P-value (significance)
PHQ: Patient health questionnaire
PHQ-D: German version of the PHQ
R^2^: Explained variance
RQ: Research question
SD: Standard deviation
SE: Standard error
t: T-value
