# Supplementary material for: The moderating effect of social resources on the link between study-related stressors and depressive symptoms among medical students in North Rhine-Westphalia, Germany – a cross-sectional study
Source: BMC Psychiatry. 2022 Aug 2;22:524. doi: 10.1186/s12888-022-04170-0 (PMC9345665; doi:10.1186/s12888-022-04170-0)
Supplement: Supplementary file 1 — Additional file 1: Table S1. Spearmans`rank correlation coefficients for mean stress. Table S2. Regression-coefficientsfor interactions of social resources on the association between mean stressorsand depressive symptoms. [file 12888_2022_4170_MOESM1_ESM.docx]

**Supplementary material**

The association of social resources on the relation between study-related stressors and depressive symptoms among medical students in North Rhine-Westphalia – a cross-sectional study

Nora Kappner^1^, Jessica Lang^1^, Anne Berthold^2^, Petra Maria Gaum^1^

^1^Institute for Occupational, Social and Environmental Medicine, RWTH Aachen University, Germany

²[Department of Health Sciences and Technology](http://www.hest.ethz.ch/), ETH Zürich, Switzerland

Corresponding author:

Nora Kappner

University Hospital RWTH Aachen

Institute for Occupational, Social and Environmental Medicine

Pauwelsstraße 30

52074 Aachen, Germany

Email: nora.kappner@rwth-aachen.de

Web: www.arbeitsmedizin.ukaachen.de

Phone: (+49) 241 80 88 88 1

Fax: (+49) 241 80 82 587

This document contains results for all hypotheses and the research question analysed with a combined mean stress predictor variable including all the three considered stressors: academic performance, clinical practice and faculty relations. Spearmans’ rank correlation coefficients for mean stress and all other considered variables in the manuscript are shown in table S-1.

Table S-1: Spearmans` rank correlation coefficients for mean stress

|  | Mean stress |
| --- | --- |
| Gender | -.25** |
| Academic performance | .79** |
| Clinical practice | .72** |
| Faculty relations | .78** |
| Depressive symptoms | .52** |
| Social support professors | -.38** |
| Social support fellow students | -.42** |
| Social identity | -.23** |
| Dual identity | -.27** |
| Status | .34** |

Note: ** p-value <.001

In relation to hypothesis 1, the linear regression shows a significant positive association between mean stress and depressive symptoms [t(2) = 7.4, β = .52, p <.001]. The whole model was also significant [F(2,160) = 30.4, p <.001, R² = .28].

Additionally to hypothesis 2 the interactions with all considered moderators are analysed for mean stress as predictor variable.

Table S-2: Regression-coefficients for interactions of social resources on the association between mean stressors and depressive symptoms

| **Moderator** | **B** | **S.E.** | *t* | *p* | *∆R^2^* |
| --- | --- | --- | --- | --- | --- |
| **Social support professor** | -3.0 | 2.2 | -1.4 | .18 | .01 |
| **Social support fellow students** | -1.5 | 2.2 | -0.7 | .50 | .002 |
| **Social identity** | -0.7 | 1.0 | -0.8 | .44 | .002 |
| **Dual identity** | -2.4 | 1.7 | -1.5 | .13 | .01 |
| **Status** | 0.1 | 0.1 | 1.4 | .18 | .01 |

Related to the research question the three-way-interaction of mean stress, ingroup status and study programme was not significant [t[6] = -0.6, B = -0.3, S.E. = 3.5, p = .92, ∆R² = .01].
